# Advances in Oral Drug Delivery Systems for Natural Polyunsaturated Fatty Acids: Enhancing Bioavailability and Therapeutic Potential

**DOI:** 10.3390/pharmaceutics17111377

**Published:** 2025-10-24

**Authors:** Matheus Felipe Zazula, Roberta Pozzan, Guilherme Anacleto dos Reis, Mônica Maciel, Thomas Horlem, Tayná Nery Banckes, Josilene Lima Serra Pereira, Ceci Sales-Campos, Luiz Claudio Fernandes, Walter José Martinez-Burgos, Katya Naliwaiko

**Affiliations:** 1 Laboratório de Plasticidade Morfofuncional, Departamento de Biologia Celular, Setor de Ciências Biológicas, Centro Politécnico, Universidade Federal do Paraná, Curitiba 81531-908, Paraná, Brazil; matheuszazula@gmail.com (M.F.Z.); monica.mm.maciel@gmail.com (M.M.); taynabanckes@ufpr.br (T.N.B.); katya@ufpr.br (K.N.); 2 Laboratório de Toxicologia Celular, Departamento de Biologia Celular, Setor de Ciências Biológicas, Centro Politécnico, Universidade Federal do Paraná, Curitiba 81531-908, Paraná, Brazil; 3 Departamento de Engenharia de Bioprocessos e Biotecnologia, Setor de Ciências Tecnológicas, Centro Politécnico, Universidade Federal do Paraná, Curitiba 81531-990, Paraná, Brazil; guireistda@gmail.com; 4 Laboratório de Metabolismo Celular, Departamento de Fisiologia, Setor de Ciências Biológicas, Centro Politécnico, Universidade Federal do Paraná, Curitiba 81531-908, Paraná, Brazil; horlem@ufpr.br (T.H.); lcfer@ufpr.br (L.C.F.); 5 Departamento de Tecnologia de Alimentos, Instituto Federal de Educação, Ciência e Tecnologia do Maranhão, Campus Maracanã, São Luís 65095-460, Maranhão, Brazil; josilene.serra@ifma.edu.br; 6 Laboratório de Cogumelos e Fungos Comestíveis, Instituto Nacional de Pesquisas da Amazônia (INPA), Campus Aleixo I, Av. André Araújo, 2936, Aleixo, Manaus 69060-001, Amazonas, Brazil; ceci.cog@gmail.com; 7 Facultad de Ingeniería, Universidad Andres Bello, Av. Antonio Varas, 880, Santiago 8370035, Chile

**Keywords:** omega fatty acids, polyunsaturated fatty acids (PUFAs), drug delivery systems, nutraceuticals, encapsulation techniques

## Abstract

Omega-3 and omega-6 fatty acids play essential roles in human health, being widely used in the prevention and treatment of various conditions, such as cardiovascular diseases, inflammation, and metabolic disorders. However, their oral administration faces significant challenges, including low solubility, rapid oxidation, and low bioavailability, which limit their therapeutic efficacy. This article explores recent advances in oral drug delivery systems designed for polyunsaturated fatty acids, highlighting how innovative technologies, such as nanoemulsions, liposomes, microencapsulation, and solid lipid nanoparticles (SLNs/NLCs), can improve their stability, absorption and clinical performance. In addition, the main natural sources of these compounds, as well as their extraction and purification methods, and the challenges related to their absorption and metabolism are discussed. This narrative review was based mainly on a comprehensive search of peer-reviewed literature published between 2015 and 2025 in PubMed, Scopus, and Web of Science. The therapeutic benefits of these emerging approaches are analyzed by comparing conventional methods with modern delivery strategies to optimize the use of omega-3 and omega-6 in the body. Finally, the article outlines future perspectives and regulatory challenges associated with these technologies, highlighting their potential to revolutionize the administration of essential fatty acids and broaden their applications in medicine and nutrition.

## 1. Introduction

Among the macronutrients in diets, lipids account for approximately 25–45% of the total calories of the diet of humans in occidental countries, with their main sources being vegetable oils and animal fats [1,2,3]. Fatty acids are structural constituents of lipids, consisting of linear hydrocarbons that contain between four and thirty-six carbon atoms and a terminal carboxyl (-COOH) functional group. Carbon chains may contain only single bonds (saturated fatty acids, SFA), one double bond (monounsaturated fatty acids, MUFA n-9 or n-7), or multiple double bonds (polyunsaturated fatty acids, PUFAs). PUFAs are particularly relevant from both nutritional and therapeutic perspectives, and their classification in different families (n-3 and n-6) is based on the position of the first unsaturation from the methyl end of the molecule [4,5,6].

Long-chain polyunsaturated fatty acids (LC-PUFAs) comprise molecules with 20 or more carbon atoms and two or more unsaturations, playing essential roles in the maintenance of cellular homeostasis, inflammatory modulation, and neurodevelopment [6,7]. The main families of LC-PUFAs include the omega-3 fatty acids (n-3) and omega-6 (n-6), which differ by the location of the first double bond at the third or sixth carbon, respectively. The n-3 PUFAs are metabolic derivatives of alpha-linolenic acid (ALA; 18:3n-3), that is converted into eicosapentanoic acid (EPA; 20:5n-3), and docosahexaenoic acid (DHA; 22:6n-3), both recognized for their anti-inflammatory, anticancer, cardioprotective, and neuroprotective properties [6,8,9,10]. Meanwhile, n-6 PUFAs are derived from linoleic acid (LA; 18:2n-6), whose endogenous conversion results in the formation of arachidonic acid (ARA; 20:4n-6), a precursor of pro-inflammatory eicosanoids [11,12].

Dietary sources vary significantly in fatty acids composition: marine oils, such as fish oil, are rich in LC-PUFA of the n-3 family, whereas vegetable oils, such as soybean and corn oils, contain high levels of n-6 PUFAs [13,14]. An imbalance in the intake between these two families, particularly an increased n-6/n-3 ratio, has been associated with unfavorable metabolic outcomes, highlighting the importance of dietary strategies and technologies that promote a more balanced intake of these essential nutrients [11,15,16,17].

LC-PUFAs play essential roles in human health by contributing to the integrity of cellular membranes, the regulation of inflammation, neuromuscular development, cardiovascular function, and energy metabolism [5,18,19,20,21,22,23]. Scientific evidence also highlights their preventive and therapeutic effects, underscoring their relevance to contemporary nutrition and pharmacology [11,24,25,26,27,28,29].

Despite the well-recognized benefits, the oral administration of these fatty acids faces significant challenges that compromise their therapeutic effectiveness. Low solubility in aqueous media, combined with high susceptibility to lipid oxidation, results in chemical instability during storage and gastrointestinal transit [30,31,32]. These factors, combined with limited absorption and extensive first-pass metabolism, result in low systemic bioavailability, thereby restricting the achievement of optimal biological efficacy [30,33].

In light of these limitations, the development of controlled-release systems emerges as a valuable strategy to optimize the delivery and efficacy of LC-PUFAs. These technologies aim to protect the compounds from degradation, improve their solubilization, and promote modulated and targeted release, enhancing absorption and reducing adverse effects. Therefore, advanced oral delivery systems represent an innovative approach to improve the therapeutic impacts of natural fatty acids, responding to current demands on functional nutrition and pharmacotherapy.

In this review, we critically examine these delivery systems, highlighting their design, functionality, and potential to enhance the bioavailability and efficacy of natural polyunsaturated fatty acids. The literature search was conducted in databases including Web of Science, Scopus, and PubMed, with a focus on peer-reviewed studies published between 2015 and 2025. Earlier seminal publications were also considered when necessary to contextualize the discussion. Data extracted from the selected studies were qualitatively analyzed and organized according to the type of delivery system, formulation strategy, physicochemical properties, and reported biological or therapeutic effects. This qualitative synthesis allowed the identification of converging evidence, technological trends, and research gaps in the field.

## 2. Sources, Extraction, and Purification of Omega-3 and Omega-6

Driven by the increasing interest in the potential physiological benefits of polyunsaturated fatty acids (PUFAs) from omega-3 and omega-6 families, the market and the scientific community are seeking diverse natural sources to meet their ever-growing demand [34].

PUFAs are widely distributed in nature, with marine, plant-based, and microbial sources being the most exploited. Fish such as salmon, tuna, and sardines are major dietary sources of EPA and DHA [35]. Despite this, fish generally store relatively low amounts of oil in their bodies, which creates a larger demand for biomass for industrial-scale PUFA production, creating both environmental concerns and the need for more sustainable production strategies [36].

To find a sustainable and economically viable alternative with a reduced environmental impact, the use of microorganisms such as microalgae, bacteria, and fungi for the production of DHA and EPA has been increasingly explored. The marine protists of the Thraustochytriidae family, for example, are recognized as excellent DHA producers [37], and species such as *Schizochytrium* spp. and *Aurantiochytrium* spp. have been widely investigated as alternative sources of PUFAs. However, high extraction and purification costs still limit their large-scale industrial application [38,39].

The extraction of omega-6 is usually associated with oils obtained from vegetable sources, such as soybean, corn, sunflower, and canola oils [40]. Because vegetable oil processing is simpler than that of fish oil, it is considered a more viable and sustainable option for fatty acid production from an industrial perspective, although the conversion of these fatty acids into EPA and DHA in the human body remains limited [41].

Table 1 provides more detailed information on the main sources of omega-3 and omega-6. It should be noted that these values are not constant, since concentrations may vary substantially, depending on factors such as genotype, physiology, environmental conditions, and the extraction and purification methods employed [42].

Once obtained, PUFAs must be extracted and purified in ways that preserve their quality and structural integrity, as well as their bioactivity [54]. Currently, the most common approaches for obtaining PUFAs rely on mechanical pressing and conventional solvent-based extractions (e.g., hexane, chloroform, ethanol) [55,56,57,58,59,60]. To improve efficiency and sustainability, researchers have focused on developing innovative methods such as enzyme-assisted extraction, supercritical fluid extraction, ultrasound-assisted extraction, and microwave-assisted extraction techniques [61,62,63,64].

Following extraction, purification processes are required to ensure their suitability for food and pharmaceutical applications. Widely employed refining processes include degumming, neutralization, bleaching, and deodorization [56]. The purification step is crucial to remove impurities and stabilize the final product [65,66,67,68]. However, care must be taken to avoid degradation and the formation of trans isomers during industrial refining and purification, particularly at high temperatures or under oxidative conditions. This structural conversion modifies the spatial configuration of double bonds, decreasing nutritional quality and potentially leading to adverse metabolic outcomes [40]. To minimize such transformations, mild processing conditions are adopted, including temperature control below 200 °C, vacuum deodorization to limit oxygen exposure, and the addition of natural antioxidants (e.g., tocopherols, rosemary extract) [58,68].

Analytical quality control methods are used to confirm the chemical stability of processed oils [40,65,69]. These include the determination of peroxide value (PV), anisidine value (AV), and thiobarbituric acid reactive substances (TBARS) to evaluate primary and secondary oxidation [69]; Fourier-transform infrared spectroscopy (FTIR) and gas chromatography (GC-FID or GC-MS) to detect possible trans isomers or breakdown products [40,69]; and fatty acid methyl ester (FAME) profiling to confirm structural integrity [65]. Such quality control analyses are critical to ensuring that omega-3 and omega-6 fatty acids retain their chemical stability and bioactive potential after processing and purification.

## 3. Challenges in the Absorption and Stability of Fatty Acids

LC-PUFAs have a high susceptibility to lipid oxidation due to the presence of multiple double bonds in their molecular structure. This characteristic causes chemical instability, making LC-PUFAs more vulnerable to degradation when exposed to environmental factors such as oxygen, radiation, temperature fluctuations, and catalytic metal ions [20,31,69]. Lipid oxidation culminates in the formation of highly reactive secondary products, including hydroperoxides and aldehydes, which may compromise the functional integrity of the lipids and exert cytotoxicity and pro-inflammatory effects. In this context, the mitigation of oxidation is essential for the preservation of stability, safety, and efficiency of the therapeutic formulation of fatty acids [70,71,72].

Degradation of LC-PUFAs is modulated by multiple physicochemical factors that accelerate the lipid oxidation process and compromise the structural integrity of these compounds. Radiation exposure, particularly in the ultraviolet range, induces the formation of reactive oxygen species (ROS), promoting peroxidation of the unsaturated bonds [70,73,74,75]. An increase in temperature acts as a catalyst, intensifying the kinetics of the oxidative reactions and facilitating the generation of free radicals. The presence of molecular oxygen represents the primary oxidizing agent, which interacts directly with the double bonds of the fatty acids, triggering reactive chains that culminate in lipid decomposition [76,77]. Furthermore, variations in environmental pH may alter the ionization state of lipids and influence the rate of the chemical reactions, affecting stability and the solubilization capacity of fatty acids [78,79]. The convergence of all these factors creates a challenging scenario for the preservation of the stability and functionality of these biomolecules in pharmaceutical and nutritional systems [70,71,72].

Peroxidation of PUFAs results in the generation of a variety of secondary oxidation products, including lipid hydroperoxides, reactive aldehydes, ketones, and epoxides, which exhibit high chemical reactivity [20,69]. These oxidized metabolites interact covalently with essential cellular macromolecules such as proteins, phospholipids, and nucleic acids, triggering biochemical and structural damage and compromising cellular functionality [80,81,82]. Chronic accumulation of these metabolic products promotes mitochondrial dysfunction, redox imbalance, and enhanced systemic inflammatory response. All these factors contribute to the progression of complex pathologies, such as cardiovascular, neurodegenerative, and metabolic diseases. Advanced delivery systems for natural fatty acids may help mitigate these detrimental effects by enhancing bioavailability and targeted action, potentially reducing oxidative stress and inflammation [83,84]. Additionally, the formation of these compounds compromises organoleptic properties and the stability of nutritional formulas, negatively impacting the therapeutic effectiveness and toxicologic safety of the formulations [70,76,77,78,79].

The absorption of oral n-3 and n-6 LC-PUFAs occurs predominantly in the small intestine, involving a sequential process of lipid emulsification mediated by bile salts, formation of mixed micelles, and enzymatic hydrolysis by pancreatic lipases, followed by enterocyte uptake [85,86,87]. However, absorptive efficiency varies significantly depending on the chemical form of the compound. Initially, the triacylglycerols (TAGs) in the form of mixed micelles are digested by pancreatic lipases, releasing monoacylglycerols (MAGs) and free fatty acids (FFAs). The MAGs are notable for their pre-digested structure, which allows direct intestinal absorption without the necessity of pancreatic hydrolysis, resulting in faster plasma peaks (Tmax = 5.5 h) compared to ethyl esters (Ees) [87,88,89]. These pharmacokinetic differences are intrinsically connected to physiological challenges, such as low aqueous solubility, susceptibility to oxidation, and interindividual variability in the availability of bile salts and digestive enzymes, factors that limit the bioavailability of conventional LC-PUFAs [85,86,89,90]. Thus, the selection of chemical form and strategic formulations emerges as a critical factor to overcome absorptive barriers and maximize the therapeutic efficacy of LC-PUFAs.

## 4. Oral Delivery Systems Applied to Omega-3 and -6

### 4.1. Nanoemulsions

The application of advanced oral delivery systems has emerged as a promising strategy to overcome the inherent limitations of omega fatty acid utilization and to potentiate their therapeutic and nutritional effects in clinical practice. Approaches such as nanoemulsions, liposomes, microencapsulation, solid lipid nanoparticles (SLNs), and nanostructured lipid carriers (NLCs) have been extensively investigated for their ability to protect these bioactive compounds from degradation, regulate their release, and enhance their absorption within the gastrointestinal tract. In the following section, the key characteristics and future perspectives of these technologies for the oral delivery of omega-3 and -6 fatty acids will be critically discussed.

Nanoemulsions are thermodynamically unstable colloidal systems composed of two immiscible phases (typically oil and water) stabilized by surfactants, with an average droplet diameter ranging from 10 to 1000 nm [91]. These fine emulsions differ from conventional emulsions by their transparency, higher specific surface area, and kinetic stability [92]. Their basic structure consists of a dispersed phase (oil) containing the lipophilic bioactive compound, such as omega fatty acids, a continuous phase (generally aqueous), and emulsifying agents that prevent coalescence [93]. Nanoemulsions can be classified as oil-in-water (O/W), water-in-oil (W/O), or multiple emulsions (W/O/W or O/W/O), depending on the intended application and the polarity of the active compound [94,95].

**Figure 1:**
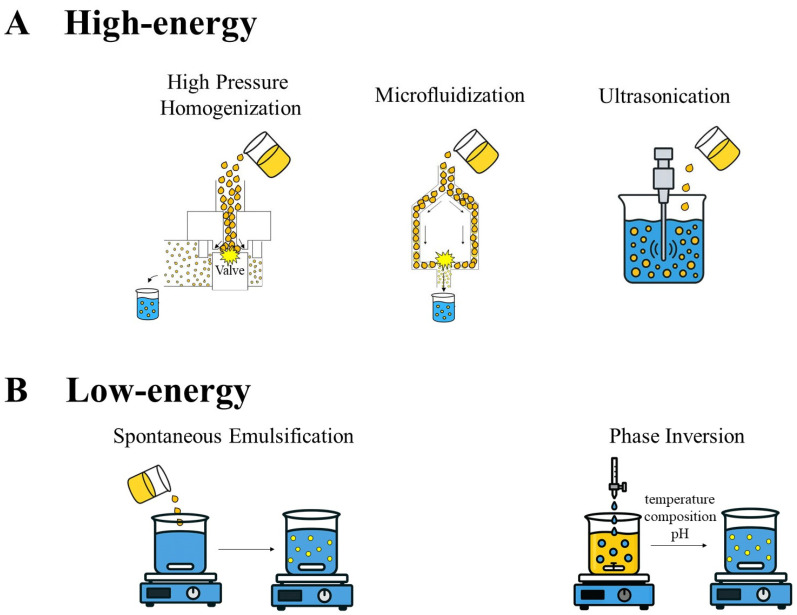
(**A**) Most commonly used high-energy processes to prepare nanoemulsions. (**B**) Most commonly used low-energy processes to prepare nanoemulsions.

Preparation methods for nanoemulsions can be classified into high-energy or low-energy processes, as shown in Figure 1 [91]. Among the high-energy methods, high-pressure homogenization, microfluidization, and ultrasonic sonication stand out, as they promote droplet disruption through intense shear forces, resulting in nanometric sizes [96,97]. These methods allow for greater control over particle size; however, they require specialized infrastructure and are energetically demanding [98,99]. In contrast, low-energy methods, such as spontaneous emulsification and phase inversion, exploit the thermodynamic properties of the system to form nanoemulsions, often eliminating the need for complex equipment [100,101].

The choice of the method depends on the nature of the fatty acid to be encapsulated, the characteristics of the oil, and the therapeutic or nutritional purpose of the formulation [102]. Spontaneous emulsification has gained prominence in the field of functional foods due to its simplicity and reduced thermal impact, thereby preserving sensitive compounds such as omega fatty acids [101,103]. However, the challenge of scaling up these techniques for industrial use still limits their large-scale application, requiring advances in process engineering and formulation [97].

The versatility of nanoemulsions lies in their ability to incorporate hydrophobic compounds, such as EPA and DHA, while protecting them from undesirable interactions with the external environment [104]. The type of surfactant (natural or synthetic), the phase ratio, and the preparation method directly influence their physicochemical properties and overall performance as a delivery system [93]. Careful selection of these components is essential to ensure both biocompatibility and system efficiency, particularly in nutritional and therapeutic applications, where safety and functionality are critical requirements [102].

The main limitation of omega fatty acids lies in their low solubility in aqueous media and their susceptibility to oxidation [105]. Nanoemulsification represents a strategic technological approach to overcome these challenges by promoting the efficient dispersion of lipophilic compounds in aqueous matrices and forming a physicochemical barrier against the effects of oxygen, pH, and temperature [106,107]. This protection is essential to preserve the functional integrity of PUFAs, whose beneficial effects on cardiovascular, neurological, and inflammatory health depend on their structural stability [108]. Furthermore, their ability to improve dispersibility in aqueous matrices and mask the residual taste of PUFAs enhances the sensory acceptability of products [109,110].

Moreover, the small droplet size in nanoemulsions facilitates intestinal absorption through both passive and carrier-mediated mechanisms, promoting paracellular and transcellular penetration and thereby enhancing the bioavailability of PUFAs [111]. Studies have demonstrated that the incorporation of these bioactive lipids into nanoemulsions results in higher plasma concentrations and more pronounced biological effects compared with conventional formulations [103,112]. Thus, nanoemulsification not only enables the oral administration of omega-3 and -6, but also amplifies their therapeutic potential. Several studies have reported the successful encapsulation of PUFAs in nanoemulsions to improve their stability and bioavailability, as shown in Table 2.

The physicochemical characterization of nanoemulsions is essential to ensure their performance as delivery vehicles [116]. System stability can be assessed using techniques such as visual inspection, zeta potential, polydispersity index (PDI), and electron microscopy, which allow the detection of phenomena such as coalescence, flocculation, and creaming [95]. In addition, droplet size and distribution can be determined by laser light scattering, fatty acid composition can be analyzed by gas chromatography, and volatile compounds associated with lipid oxidation can be quantified by GC-HS (Headspace Gas Chromatography), all of which enable a comprehensive overview of nanoemulsion behavior [119]. These characterization techniques are essential not only for evaluating the structure and performance of the delivery system, but also for confirming that the chemical integrity and oxidative stability of encapsulated PUFAs are preserved after processing.

The release kinetics of encapsulated PUFAs also represent a critical parameter, as they directly influence their bioavailability and therapeutic efficacy [106]. In vitro assays in simulated gastric and intestinal fluids allow estimation of the controlled release profile of lipophilic compounds [120]. Ideally, release should be modulated to protect fatty acids during gastric transit and facilitate their release in absorptive regions of the intestine [115].

Despite their evident advantages, nanoemulsions still face significant limitations for full commercial adoption and application. The high cost of production, particularly when involving high-energy equipment or food-grade natural emulsifiers, is a major obstacle [121]. In addition, the scalability of laboratory processes to industrial production remains a technical challenge, with risks of losing control over particle size and stability during scale-up [95].

Another critical aspect concerns long-term stability [116]. Solutions such as the addition of antioxidants, polymeric coatings, or partial refrigeration have been investigated, but these strategies are not always economically feasible [115]. Therefore, the development of more robust and cost-effective formulations is essential to enable the practical implementation of these technologies in commercial applications.

In this context, hybrid emulsions have emerged as an innovative response to the classical limitations of nanoemulsions [122]. These structures combine different materials (e.g., natural polymers, structured lipids, and solid nanoparticles) into multiphasic systems designed to enhance stability, control release, and improve the functionality of encapsulated bioactives [109]. For instance, liposome–nanoemulsion hybrid systems have demonstrated greater oxidative resistance and sustained DHA release under simulated gastrointestinal conditions, representing an innovative approach to overcome the drawbacks of conventional formulations [122].

These innovations also enable the targeted delivery of bioactive compounds through the use of functional ligands or recognition biomolecules, an approach that has been explored in nanoemulsion-based systems to improve therapeutic efficacy [122]. A recent study, for example, reported the use of folate-functionalized lipid nanoemulsions for the co-delivery of DHA and paclitaxel to tumor cells, an approach that combines controlled release with site-specific targeting and broadens therapeutic applications [118]. Nonetheless, regulatory and technological standardization challenges remain to be addressed to ensure safety and efficacy in both clinical and food-related applications [92].

Thus, nanoemulsions represent an attractive strategy for the incorporation of omega fatty acids into functional foods and therapeutic agents [96,114], and may be particularly valuable for populations with special needs, such as patients with malabsorption syndromes, inflammatory bowel diseases, or neurological disorders [120,123]. In this way, nanoemulsions are consolidated as a strategic interface between food science, pharmaceutics, and precision nutrition [124].

### 4.2. Liposomes

Liposomes are delivery systems composed of one or more concentric lipid bilayers that enclose internal aqueous compartments [108]. Their organization mimics cellular membranes, providing high biocompatibility and versatility for encapsulating both hydrophilic and lipophilic compounds [125]. Their typical composition includes natural or synthetic phospholipids and, in frequent cases, cholesterol, which stabilizes the structure and modulates membrane fluidity [126]. Liposome morphology can range from unilamellar vesicles (ULVs) to multilamellar vesicles (MLVs), directly influencing their pharmacokinetic behavior and encapsulation capacity [127].

**Figure 2:**
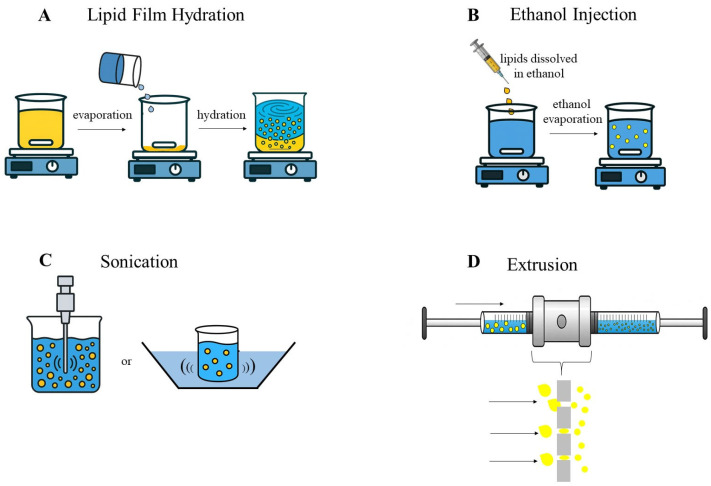
Most commonly used processes to prepare liposomes. (**A**) Lipid film hydration; (**B**) Ethanol injection; (**C**) Sonication, with sonicator or ultrasonic bath; (**D**) Extrusion, with an extruder apparatus.Arrows indicate the flow of lipid vesicles through the membrane during extrusion.

Several techniques are employed for liposome production, with the most common being lipid film hydration, ethanol injection, sonication, and extrusion (Figure 2) [128,129]. The choice of production method directly affects particle size, distribution, lamellarity, and encapsulation efficiency [126]. In addition, the loading strategy (direct, remote, or dual) also influences the localization of the active compound within the liposome (bilayer vs. core) and, consequently, the release kinetics, allowing modulation from fast to sustained profiles [130].

The characterization of these structures is highly recommended for their validation and can be carried out through physicochemical analyses such as spectroscopy (FTIR, UV-vis), dynamic light scattering (DLS) for particle size determination, electron microscopy (TEM or SEM) for morphological assessment, and chromatographic or spectrophotometric techniques to determine encapsulation efficiency and release kinetics [131]. Thermal, oxidative, and long-term stability assays are also essential to ensure a robust formulation, particularly in systems containing omega-3, which are highly susceptible to degradation [132]. In addition to assessing structural parameters, these techniques are fundamental for verifying that omega fatty acids retain their chemical stability and remain protected from oxidative degradation throughout the liposomal formulation and storage process.

The functionality of liposomes arises from their lamellar architecture, which enables the protection of sensitive molecules against chemical, enzymatic, or oxidative degradation [131,133]. In the context of omega fatty acid delivery, their lipid bilayer serves as an effective matrix for incorporating these unstable compounds, contributing to their solubilization, protection, controlled release, and masking of taste and odor [129]. The encapsulation efficiency of PUFAs depends on liposome membrane fluidity, the nature of the lipids employed, and the preparation technique used, with optimized formulations achieving rates above 50% [134,135].

This capability is crucial for addressing the intrinsic oxidative instability of unsaturated fatty acids, as it can extend the shelf life of these biomolecules [128]. In particular, omega-3 and -6 fatty acids can integrate into the hydrophobic region of the liposomal bilayer, where they are protected from direct exposure to the aqueous environment and from oxidative, hydrolytic, and thermal degradation, thereby significantly contributing to the preservation of their biological activity [136,137].

The amphiphilic nature of liposomes favors interactions with biological membranes, promoting high cellular uptake and enabling modulation of the biodistribution profile of the encapsulated actives [127]. Thus, among the main benefits of liposomes in omega fatty acid delivery is their ability to modulate the release of the active compound over time or in response to specific stimuli (e.g., pH, temperature), allowing targeted release in physiologically relevant environments such as the intestine [138,139,140]. This controlled release may also prevent systemic concentration peaks, thereby reducing side effects and optimizing therapeutic efficacy [141].

Another relevant aspect is the possibility of liposomal surface functionalization with specific ligands (e.g., peptides, polysaccharides, antibodies), which can be applied to promote active targeting to specific tissues or cells and to provide greater protection against thermal and oxidative instability [142,143]. Alternatively, functionalization can also be achieved through the co-encapsulation of bioactive compounds with complementary properties, as demonstrated by Ref. [134], who encapsulated curcumin and omega-3 simultaneously in nanoliposomes, obtaining enhanced antioxidant and antimicrobial activity due to the synergistic effect of the two compounds.

Extensive research has investigated the incorporation of omega fatty acids into functionalized liposomes for specific pharmaceutical and nutritional applications [105,107]. The incorporation of liposomes into functional foods represents a technological frontier with great potential, particularly for products enriched with omega-3 [144,145,146]. Several studies evaluating the application of liposomes in the encapsulation of omega fatty acids are summarized in Table 3.

Despite significant advances, liposomes still face important practical limitations. Large-scale production remains challenging, partly due to the complexity of preparation methods and the need for rigorous purification [125]. The high cost of pharmaceutical- or food-grade lipids, particularly natural phospholipids, also restricts the industrial application of liposomal formulations in low-cost functional foods and supplements [108,148].

In addition, long-term stability is a critical issue, especially for liposomes containing unsaturated fatty acids [149]. Vesicle fusion, lipid oxidation, and premature release of the active compounds are recurring challenges, often requiring the addition of antioxidants, cryoprotectants, and pH adjustments in the formulation [150]. Strategies such as liposome lyophilization, although effective, further increase production costs [139,151]. Therefore, the development of more stable, scalable, and cost-effective formulations is a priority for advancing liposome applications in this context.

Recent innovations in the field of liposomes have focused on the development of modified systems, such as PEGylated liposomes, liposomes functionalized with specific biomolecules, and nanoliposomes, which offer improved tissue penetration and enhanced kinetic stability [137,150,152]. These modifications aim to overcome traditional limitations related to stability, systemic half-life, and release control.

In particular, nanoliposomes have been studied as effective vehicles for the oral delivery of omega-3, showing encouraging results regarding intestinal absorption and their impact on inflammatory and lipid biomarkers, opening new perspectives for the clinical and nutritional use of fatty acids [138]. One example is the clinical trial conducted by Ref. [147], in which PEGylated nanoliposomes of unsaturated pistachio oils (rich in EPA and DHA) were administered to patients with multiple sclerosis, resulting in increased serum fatty acid levels, reduced inflammatory cytokines, and significant clinical improvement [147].

Thus, in the context of clinical nutrition and pharmacology, liposomes represent an ideal system for the targeted and efficient delivery of essential fatty acids in patients with specific needs, such as malnutrition, chronic inflammatory diseases, neurological disorders, or cardiovascular conditions [141,147]. Nevertheless, progress in the regulation and standardization of these systems is critical to ensure their widespread adoption in clinical and commercial settings [132].

### 4.3. Microencapsulation

Microencapsulation is the most widely used technique for entrapment systems of PUFAs. The process consists primarily of entrapping droplets of oils with a high content of omega fatty acids in polymeric matrices, such as polysaccharides, proteins, lipoproteins, glycolipids, or a mixture of these [153].

Microencapsulation of highly unstable fatty acids protects them against oxygen, radiation, heat, and traces of metals that induce or accelerate oxidation or degradation processes. It also serves to mask their unpleasant odors and flavors, making the formulations more sensorially acceptable. In addition, it provides controlled or targeted release, either in the gastrointestinal tract or as enteric release (only at intestinal pH), which increases bioavailability and efficacy [154,155].

Different types of materials have been used for the microencapsulation of these compounds, which are selected depending on their purpose, whether to serve as a barrier to prevent oxidation, or to achieve a certain type of release. These materials are classified into four main groups: biopolymeric, lipid, synthetic biodegradable polymers, and auxiliary functional compounds [156]. The first group is subdivided into polysaccharides, cellulose, and proteins. Table 4 presents the different materials used in the microencapsulation of omega fatty acids as well as their properties and applications.

Due to the highly reactive chemical nature of these lipids, the use of a single encapsulating material does not always provide adequate protection. For this reason, the combination of different materials has been explored to develop of more effective and resistant release systems. Since mixtures of materials act synergistically, they allow the use of the complementary properties of each component to improve their function. Polysaccharides such as pectins, alginates, gum arabic, and modified starches provide important structural characteristics, such as gel formation, resistance to gastric acidity, and diffusion control. Protein materials confer them emulsifying and film-forming properties, in addition to stabilizing interfaces and, in the case of lipids, forming highly efficient hydrophobic barriers, significantly reducing the penetration of oxygen and water [157,158].

Among the most efficient systems that have been used for the microencapsulation of PUFAs are mixtures of gum arabic and maltodextrin in proportions 1:1 or 3:2, used mainly for the encapsulation of fish and flaxseed oils, combined with spray drying encapsulation method [159,160]. Another system used in the microencapsulation of fish and krill oil is the mixture of gelatin and gum arabic in a proportion of 1:1 via coacervation. However, for the coacervation process to occur efficiently, the pH of the complex must be between 4.0 and 4.2 and the temperature between 40 and 50 °C [161]. The alginate–chitosan system in proportions 8:2 via ionic gelation is mainly used for the production of gastroprotective capsules in the microencapsulation of linseed oil [162,163].

**Figure 3:**
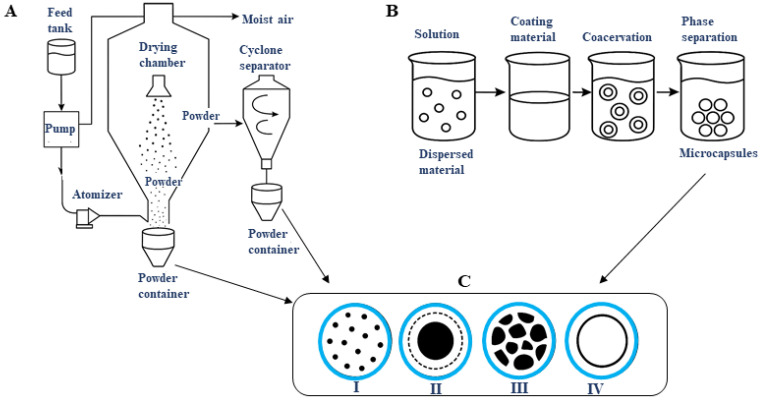
(**A**) Microencapsulation process via spray drying; (**B**) General process of coacervation for microencapsulation; (**C**) Microcapsule structures that can be generated by spray drying and coacervation, (I) matrix particles; (II) core–shell structures; (III) multicore or polynuclear capsules; (IV) empty core (vacuolated) capsules.

In general, the most commonly used operations in microencapsulation are spray drying and coacervation. In spray drying, liquids and emulsions are transformed into dry powders. The system generally consists of a feed tank containing the solution or emulsion, a feed pump that transfers the solution to the atomizer, which transforms the liquid into droplets, and the drying chamber, where the droplets come into contact with hot air, evaporating the water. The system also has a hot air inlet, generally at the top, a cyclonic separator that separates the solid material carried by the air, and collectors for the encapsulated material (Figure 3A) [164]. In the case of coacervation, a phase separation process occurs, in which two immiscible liquids form a phase rich in the coacervate. It is generally a process that occurs in four stages: (I) solution, where initially the material to be encapsulated is dispersed in a liquid phase; (II) coating material, in which one or a mixture of polymers is added to the solution, responsible for forming the walls of the microcapsules; (III) coacervation, where the system undergoes changes in pH and temperature, promoting phase separation; (IV) separation, where the capsules are stabilized (by cooling, crosslinking, or drying) (Figure 3B).

Four types of microcapsule structures can be generated via spray drying or coacervation, based on the organization of the core and wall. (I) matrix particles, where the active agent is dispersed throughout the matrix of the encapsulating material, generally applied when gradual controlled release is required. (II) core–shell structures, where the active agent is surrounded by a continuous wall, making them efficient against oxidation and effective in targeted release processes; (III) multicore or polynuclear capsules, where several cores are surrounded by a single wall—these are relevant in simultaneous release processes; (IV) empty core (vacuolated) capsules, which have a central cavity with a thin wall (Figure 3C). The center may be empty or contain volatile gas/fluid, so the release occurs explosively [165]. In the case of spray drying, the structures I, II, and IV are generally formed, and in coacervation, structures I and III predominate.

A comparative study between different fish oil microencapsulation techniques (spray drying (SD), spray freeze-drying (SFD), freeze-drying (FD) and microwave freeze-drying (MFD)) revealed significant variations in the encapsulation efficiency and functional properties of the microcapsules [166]. The SD technique presented the highest encapsulation efficiency (86.98%), highlighting its high lipid-loading capacity, excellent visual appearance, and good powder flowability. However, it showed slightly lower oxidative stability than the other lyophilization-based techniques. SFD, with an efficiency of 77.79%, stood out for providing microcapsules with better solubility, being suitable for applications in aqueous systems. The FD and MFD techniques presented lower efficiencies (63.29% and 57.89%, respectively), but are recognized for favoring oxidative stability, being useful in formulations where the preservation of unsaturated fatty acids is critical.

To assess whether the chemical structure of microencapsulated PUFAs is preserved, analytical techniques such as gas chromatography, FTIR, and oxidative stability assays (e.g., peroxide value and TBARS) are commonly applied after processing and during storage, particularly in formulations subjected to high temperatures such as spray drying [156,157].

### 4.4. Solid Lipid Nanoparticles (SLNs) and Nanostructured Lipid Carriers (NLCs)

SLNs and NLCs are advanced lipid-based delivery systems. The former are solid lipid nanoparticles stabilized by surfactants that function as delivery systems (carriers) for bioactive compounds, especially those of a lipophilic nature, such as omega fatty acids. These nanoparticles have diameters between 10 and 1000 nm and are composed of lipids that remain solid at temperatures between 25 and 37 °C, such as long-chain triglycerides, waxes, and saturated fatty acids. In general, the surface of the nanoparticles is stabilized by emulsifiers or surfactants such as lecithin, stearates, and polysorbates [167,168].

**Figure 4:**
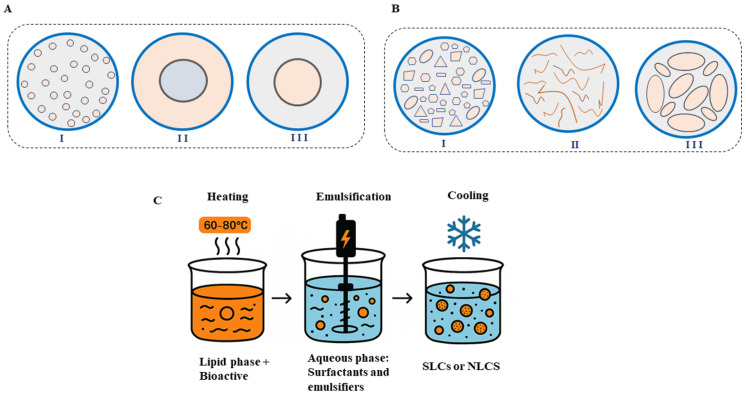
Structural types of lipid-based nanocarriers: (**A**) solid lipid nanoparticles (SLNs)—(I) homogeneous matrix, (II) drug-enriched shell, and (III) drug-enriched core; (**B**) nanostructured lipid carriers (NLCs)—(I) imperfect matrix, (II) amorphous matrix, and (III) multiple-chamber matrix. (**C**) General process for obtaining SLCs and NLCs.

SLNs are classified into three categories (I, II, and III) based on the internal structure of the lipid matrix, which influences the encapsulation capacity, the stability of the bioactive compound, and the release profile. In type I, the homogeneous matrix and the bioactive molecules are dispersed in the lipid matrix. In type II, the solid lipids crystallize first, concentrating the drug in the outer layer. And in type III, the core is rich in the bioactive compound and is surrounded by a lipid layer (Figure 4A) [169].

NLCs are also nanoparticles, with diameters between 50 and 300 nm, formed by lipid mixtures. They belong to the second generation of lipid nanoparticles, developed to overcome some limitations of SLNs, such as low active ingredient loading capacity and premature release. However, NLCs systems face challenges such as complex formulations, stability, and biological barriers [170,171].

NLCs are also classified into three categories. Type I, or imperfect matrix, is characterized by presenting imperfections in the crystalline structure generated by the mixture of solid and liquid lipids, resulting in a greater loading capacity. Type II, or amorphous matrix, occurs when the lipid matrix does not form a crystalline structure after solidification but remains amorphous, without a defined structural order. Type III, or multi-chamber matrix, consists mainly of a heterogeneous mixture of lipids in solid and liquid state, forming distinct compartments inside the particle. This configuration allows the co-encapsulation of compounds with different polarities, promoting controlled release and providing highly useful, especially in multifunctional formulations (Figure 4B) [167,172].

In general, the production process for each system occurs in five stages. Initially, heating is carried out above the melting point temperature of solid lipids (SLNs) or solid and liquid lipids (NCLs), generally between 60 and 80 °C, where a homogeneous lipid phase is obtained. The bioactive compound is solubilized in this same system. At the same time, the aqueous phase is prepared, containing surfactants and emulsifiers, including polysorbates and lecithins. This phase is also heated to the same temperature to avoid thermal shocks within the system. The emulsion is then produced by mixing the two phases, that is, by adding the lipid phase to the aqueous phase under high-speed stirring until an oil-in-water emulsion is formed with fine lipid droplets dispersed throughout the system. Finally, the emulsion is rapidly cooled, promoting the solidification of the most saturated lipids and the formation of nanoparticles (SLNs or NLCs), which are dispersed in the aqueous medium (Figure 4C) [173]. To verify whether these encapsulation process preserves the chemical stability of omega fatty acids, analytical techniques such as gas chromatography, FTIR, and oxidative stability tests (e.g., peroxide value and TBARS) are typically employed after nanoparticle formation [173,174].

Ref. [174] developed solid lipid nanoparticle (SLN) systems for the encapsulation of fish oil, with a formulation consisting of glyceryl distearate (solid lipid), Poloxamer 407 (emulsifier), and fish oil rich in omega-3 fatty acids, with the addition of 100 ppm of α-tocopherol as a bioactive compound. The preparation of the system involved hot homogenization followed by ultrasonic sonication. The particles obtained had an average size of approximately 119 nm and a polydispersity index between 0.12 and 0.17, indicating a homogeneous size distribution. Compared to conventional emulsions, SLNs containing α-tocopherol demonstrated greater protection against oxidation, prolonging stability without compromising sensory quality of the product. In addition, the system presented significantly superior protection against primary and secondary oxidative processes.

Ref. [174] also prepared two types of SLNs containing the bioactives docosahexaenoic acid (DHA) and α-linolenic acid (ALA). Both SLNs were formulated with resveratrol stearate, melted together with the bioactives at temperatures between 60 and 65 °C. The aqueous phase was composed of Tween-80 and 1-butanol, also heated in the same temperature range. The two phases were mixed until the formation of a transparent microemulsion, which was quickly poured into water at 2 °C, in a ratio of 1:20 (% *v*/*v*), under vigorous stirring (8000 rpm for 15 min). Then, the system was filtered and washed using 100 kDa membranes to remove residual nanoparticles and impurities. The average sizes of DHA and ALA SLNs were 1000 and 842 nm, respectively.

These nanoparticles were tested in vitro in human colorectal cancer cells, specifically adenocarcinoma (HT-29) and carcinoma (HCT116). SLNs increased the cellular uptake of DHA and ALA by approximately 277% and 223%, respectively, compared to their free forms. Furthermore, they demonstrated significant inhibition of cell proliferation in both cancer cell lines. At concentrations of 25 μM, SLNs-DHA inhibited approximately 60–65% of cell proliferation, while SLNs-ALA showed inhibition between 45 and 50%. These values were considerably higher than those obtained with free fatty acids, whose inhibition did not exceed 35%, indicating that SLNs significantly enhanced the antiproliferative effect of the bioactives.

Ref. [175] developed lipid nanoparticles of the NLC type (nanostructured lipid carriers) containing DHA, to prolong the release of the active ingredient, improve its antioxidant stability, and enhance its anti-inflammatory action in vitro. The lipid phase of the NLCs was composed of Compritol^®^ 888 ATO and refined fish oil, while the aqueous phase contained Tween-80 and soy lecithin. Both phases were heated to 70 °C, subsequently emulsified, sonicated for 10 min (400 W), and then cooled under stirring. The average size of the NLCs obtained was approximately 164 nm.

It is noteworthy that the NLCs showed greater free radical neutralization capacity compared to DHA in its free form. In vitro biological assays performed with RAW 264.7 macrophages stimulated with *Escherichia coli* O111:B4 lipopolysaccharide demonstrated that treatment with NLCs-DHA promoted a significant reduction in the inflammatory response. Compared to free DHA, NLCs reduced by approximately 60% the production of IL-6 (interleukin-6), from 800 to 320 pg/mL, and of TNF-α (tumor necrosis factor alpha), from 1200 to 480 pg/mL. Furthermore, the relative expression of the COX-2 (cyclooxygenase-2) enzyme was reduced from 1.0 to 0.4, demonstrating the superior anti-inflammatory potential of the nanostructured formulation.

## 5. Therapeutic Applications, Clinical Benefits, and Possible Adverse Effects

LC-PUFAs, especially n-3 (EPA and DHA) and n-6 (ARA), perform crucial functions in physiological homeostasis and in the treatment of multiple pathologies. These structural lipids modulate the fluidity and permeability of the plasma membrane, influence the formation of lipid microdomains, and are involved in intracellular signaling through the generation of eicosanoids, resolvins, and protectins, which regulate inflammatory response and promote the resolution of the inflammatory process [176,177,178]. Additionally, these fatty acids exert direct effects on gene expression and epigenetic modulation of inflammation, acting as ligands for nuclear receptors such as PPARs (Peroxisome Proliferator-Activated Receptors), which reinforces their central role in metabolic and immunological regulation [21,179].

Immunomodulatory, neuroprotective, and antioxidant properties of LC-PUFAs are extensively investigated in a clinical context, demonstrating their potential in mitigating cardiovascular, neurodegenerative, autoimmune, chronic inflammatory, and metabolic diseases. Thus, omega fatty acids are regarded as strategic biomolecules for advanced therapeutic interventions, with a significant impact on the modulation of complex pathophysiological processes [20,25,176]. These lipid mediators not only modulate inflammation but also play a key role in cardiovascular protection, blood pressure regulation, lipid metabolism control, and prevention of endothelial dysfunction. In elderly populations, neuroprotective and antioxidant effects of LC-PUFAs contribute to the preservation of cognitive function and the attenuation of degenerative processes associated with aging [11,12,25]. In addition, they have been associated with anticancer effects, mainly through the modulation of inflammatory signaling, cell proliferation, and apoptosis pathways, highlighting their broader therapeutic potential [6].

LC-PUFA plays a pivotal role in physiology and the therapeutic management of metabolic diseases, including insulin resistance, obesity, and the regulation of energy metabolism [6,178]. These lipids modulate cellular signaling at the molecular level, influencing cascades such as PPAR-α and PPAR-γ pathways, the NF-κB pathway, and adipokine-mediated signaling, resulting in the attenuation of chronic inflammation, which characterizes insulin resistance [180,181,182,183]. Beneficial effects include the activation of AMP-activated protein kinase (AMPK), inhibition of hepatic lipogenesis, and stimulation of beige adipocyte differentiation, which contributes to adaptive thermogenesis and the control of visceral adiposity [184,185].

Omega-3 fatty acids promote improvements in plasma lipid profile, activation of mitochondrial β-oxidation, and the induction of thermogenesis through the regulation of UCP-1 (uncoupling protein) expression in brown adipose tissue, contributing to the reduction in adiposity and the improvement in energy homeostasis [184,186,187,188]. The influence of these lipids on metabolically active organs, such as the liver, skeletal muscle, and adipose tissue, demonstrates their multifaceted therapeutic potential, mitigating complex metabolic dysfunction associated with obesity and type 2 diabetes. However, interindividual heterogeneity in the response to these compounds underscores the need for a personalized approach, considering genetic polymorphisms and the gut microbiome as key modulators of therapeutic efficacy [189].

Furthermore, accumulated evidence suggests that LC-PUFAs positively modulate aging-associated processes, including oxidative stress, chronic low-grade inflammation (inflammaging), and loss of muscle mass. EPA and DHA promote antioxidant gene expression and the preservation of mitochondrial integrity, contributing to the maintenance of cellular functionality in the elderly [190,191,192]. Experimental models also indicate that n-3 supplementation could delay sarcopenia and cognitive decline.

However, some authors point out that excessive n-3 intake may cause adverse effects similar to those resulting from high levels of n-6 present in modern Western diets, including increased inflammatory processes and oxidative damage, weight gain, and metabolic and structural changes [193,194]. There are already studies in the literature demonstrating a reduction in the potential beneficial effect of supplementation with EPA and DHA in humans regarding the risk of cardiovascular diseases [8], blood pressure [29], and diabetes [195]. There is also evidence that excessive n-3 intake may induce lipid peroxidation, especially when antioxidant intake is insufficient, exacerbating systemic oxidative stress.

The regulation of specific dosages for n-3 and n-6 fatty acids in nutritional supplements remains fragmented and insufficiently rigorous at the global level, which leads to relevant clinical implications [15,196]. Different regulatory agencies around the world adopt heterogeneous criteria for the recommendation and control of EPA and DHA concentrations in commercial formulations, reflecting the inherent complexity involved in assessing their bioavailability, efficacy, and safety [197,198]. The absence of standardization limits precise prescription and therapeutic monitoring, increasing the risk of underdosage, which may compromise the expected beneficial effects, or overdosing, which is associated with potential adverse effects, such as coagulation disorders and drug interactions.

Toxicity associated with excessive consumption of LC-PUFAs is expressed through adverse changes in lipid profile, including an increase in oxidized low-density lipoprotein (oxLDL), known as a critical pro-atherogenic factor promoting oxidative stress, endothelial dysfunction, and chronic vascular inflammation [199,200,201]. An imbalanced intake of n-6 and n-3 fatty acids may impair the physiological synthesis of eicosanoids derived from ARA and EPA, disrupting the regulation of inflammatory responses and favoring persistent pro-inflammatory states that negatively influence systemic metabolism [11,12,25,202]. Moreover, there is evidence that the excessive intake of LC-PUFAs may alter the cellular membrane fluidity and negatively modulate nuclear receptors involved in metabolic homeostasis, such as PPARs, amplifying metabolic and cardiovascular risks [203,204].

Considering interindividual variability in absorption, metabolism, and pharmacodynamic effects of LC-PUFAs, individualized clinical monitoring is crucial for the safety and efficacy of the treatment. Periodic evaluation of lipid biomarkers, coagulation parameters, inflammatory markers, and immunological function allows precise dosage adjustments, minimizing risks and enhancing therapeutic benefits. Furthermore, the evaluation of the n-6/n-3 fatty acid ratio and antioxidant status helps to optimize therapy, preventing complications associated with oxidative stress and metabolic disorders. Thus, the personalization of omega fatty acid therapy emerges as an indispensable requirement for the safe and effective use of these compounds in contemporary clinical practice [189,205,206].

Conventional LC-PUFA supplementation forms, such as oil capsules or simple emulsions, have many limitations that may compromise the stability, bioavailability, and therapeutic efficacy of the compounds. As previously mentioned, the high lipid oxidation susceptibility, low aqueous solubility, and gastrointestinal degradation reduce the absorption efficiency and increase the variability of the clinical response [20,71]. Conversely, innovative release systems, such as solid lipid nanoparticles, nanoemulsions, and liposomes, have demonstrated in pre-clinical and clinical studies significant advantages, such as increased chemical stability, facilitated enteric absorption, controlled release, and targeted delivery to specific tissues. These strategies have been associated with improved outcomes in models of inflammation, neuroprotection, metabolic dysfunction, and immunomodulation, demonstrating their potential as next-generation therapeutic platforms [207,208].

The development of advanced delivery systems for fatty acids necessitates the re-evaluation of classical dose parameters, routes of administration, and dosing frequency. Technologies such as functionalized nanoparticles enable the use of significantly lower doses with equal or even greater efficacy, promoting greater intestinal retention, permeability, and protection against enzymatic degradation [207,209,210]. In addition, those supplement formulations offer alternatives to the oral route, such as transmucosal, intranasal, and parenteral delivery, enhancing clinical applicability in patients with impaired gastrointestinal absorption. The frequency of dose administration can also be improved with sustained-release systems, reducing plasma fluctuations and enhancing patient treatment adherence [211,212,213,214]. Such innovations must, however, be accompanied by rigorous pharmacokinetic and pharmacodynamic analyses to ensure reproducibility and therapeutic safety [215,216].

Nanomaterials and intelligent release systems for fatty acids require detailed toxicological and pharmacokinetic analyses, as properties such as particle size, surface charge, and biodegradability may significantly influence their interactions with tissues and biological barriers. In vitro and in vivo studies have demonstrated that physiological lipid-based platforms, natural polymers (e.g., chitosan and alginate), and biocompatible surfactants exhibit low cytotoxic potential and good tolerability [217,218]. Moreover, pharmacokinetic profiles reveal improvements in plasma half-life, area under the curve (AUC), and bioavailability without undesirable tissue accumulation. Nonetheless, extrapolation to humans requires well-designed clinical studies and compliance with international guidelines (e.g., FDA—Food and Drug Administration, EMA—European Medicines Agency, and ICH—International Council for Harmonization) for risk–benefit evaluation [218,219,220].

Although the clinical literature on novel fatty acid delivery systems is promising, it continues to exhibit considerable methodological heterogeneity. Many studies have small sample sizes, lack a placebo group, and have short follow-up durations, which limit the robustness of the findings. Even though there is consistent evidence that nanoparticles and nanostructured emulsions enhance the bioavailability of EPA and DHA, with superior effects on inflammatory markers, lipid profile, and cognition in specific populations [220,221,222], there is still a lack of multicenter, randomized, long-term clinical trials directly comparing conventional supplement formulations with advanced delivery forms. Standardization of protocols, dose, and clinical endpoints is essential to strengthen the evidence and guide clinical practice grounded in translational science [192,222].

Innovations in fatty acid delivery systems demonstrate high translational potential, which is particularly relevant given the growing demand for more effective and personalized nutraceutical therapies. Driven by ongoing advances in nanotechnology, bioengineering, and artificial intelligence applied to the design of lipid vectors, it is anticipated that the integration of these innovative platforms into clinical practice will steadily increase over the next few years, particularly in the context of cardiovascular and neurodegenerative diseases as well as chronic metabolic and inflammatory disorders. Regulatory validation, in conjunction with industrial-scale manufacturing processes that guarantee stability and reproducibility, will be critical for enabling the broad clinical adoption of these technologies. Moreover, the integration of comprehensive omics datasets, predictive biomarkers, and pharmacogenomic analyses will further support the development of safer and more personalized applications of these emerging platforms.

## 6. Conclusions

In recent decades, the field of natural fatty acid delivery systems has experienced remarkable progress, driven by innovative interdisciplinary approaches. Hybrid bioengineering systems that integrate lipid nanoparticles with intelligent materials have stood out by achieving significant progress in safeguarding against oxidative degradation, controlled release, and targeted delivery to specific sites. Targeted delivery technologies utilize surfaces functionalized with specific ligands—such as antibodies, peptides, and aptamers—that recognize receptors on target cells, thereby enhancing selectivity and therapeutic efficiency while limiting systemic adverse effects. Furthermore, the integration of artificial intelligence and machine learning algorithms into the formulation and development processes facilitates predictive modeling of pharmacokinetics and pharmacodynamic interactions, optimizing physicochemical and biopharmaceutical parameters, and accelerating the personalization and scalable deployment of delivery systems.

Despite substantial technological advancements, the translation of these innovations into clinical and commercial applications remains challenging due to stringent regulatory requirements and standardization difficulties. The evaluation of toxicological safety, therapeutic effectiveness, and physicochemical stability must be conducted through robust, validated protocols that meet criteria established by regulatory agencies, including the FDA, EMA, and ICH. The complexity of hybrid systems demands advanced analytical methods for structural characterization, quality control, and batch-to-batch assurance, which are essential for industrial scalability. Furthermore, the absence of well-defined and harmonized regulatory frameworks for natural lipid-based delivery systems poses a significant regulatory challenge. Addressing this barrier requires the coordinated development of integrated initiatives among academic institutions, industry stakeholders, and regulatory authorities, aimed at formulating clear, effective, and internationally harmonized regulatory guidelines that facilitate innovation, ensure product safety, and promote global market access.

In summary, the continuous advancement in natural fatty acid delivery systems is crucial for overcoming the intrinsic challenges of stability and bioavailability, thereby enabling broader therapeutic and nutritional applications. The development of novel delivery strategies that merge nanotechnology, functional biopolymers, and computational tools contributes to the evolution of lipidomic pharmacotherapy, promoting more effective, safer, and more specific formulations. This multidisciplinary convergence establishes an innovative paradigm that integrates materials science, biotechnology, and artificial intelligence, setting the stage for the future of pharmaceutical fatty acid delivery, aligned with the current clinical, technological, and regulatory demands of the field.

## Figures and Tables

**Table 1:** The different sources of omega-3 and -6.

Type	Source	Omega-3 (%)	Omega-6 (%)	References
Vegetable	Flaxseed oil	65.84 (ALA)	16.39 (LA)	[43]
Vegetable	Chia oil	63.64 (ALA)	19.84 (LA)	[44]
Vegetable	Canola oil	11 (ALA)	21 (LA)	[45]
Vegetable	Olive oil	19.47 (ALA)	17.93 (LA)	[46]
Vegetable	Walnuts	17.9 (ALA)	63.8 (LA)	[47]
Animal	Salmon	3–4 (EPA) 9–12 (DHA)	-	[48]
Animal	Sardine	17.3–23.7 (EPA) 5.82–13.5 (DHA)	-	[49]
Animal	Mackerel	4.93–5.81 (EPA) 12.56–15.01 (DHA)	-	[50]
Animal	Tuna	9.32–9.56 (EPA) 18.76–25.88 (DHA)	-	[51]
Animal	Trout	3.65–5.54 (EPA) 13.53–32.81 (DHA)	-	[52]
Microbial	*Schizochytrium* sp.	35–40% (DHA)	-	[37]
Microbial	*Aurantiochytrium* sp.	25.98–35.76 (DHA)	-	[39]
Microbial	*Mortierella alpina*	-	46.9–66.4 (ARA)	[53]

**Table 2:** Encapsulation of Omega Fatty Acids in Nanoemulsions.

Type of Omega	Source of Omega	Stabilizer/Matrix	Production Method	Droplet Size	Additional Remarks	References
ALA	Soybean and walnut oil	Xanthan gum (in nanoemulgel) + inulin	Microfluidization	~138 nm	Greater stability with lower oil/gum ratio (1:3); gel-like behavior; high viscosity	[98]
EPA and DHA	Fish and flaxseed oil	Tween 80	Spontaneous emulsification + High-pressure homogenization	<130 nm	Development of topical gel for psoriasis treatment in mice; ↑ skin permeation (1.3–1.4×) and dermal retention; ↓ TNF-α and IL-6; ↓ PASI	[113]
ALA	Flaxseed oil (3%)	Tween 80 (28.97%) + Span 80 (7.03%) + ethanol (10%)	Low-energy method (HLB)	~60 nm	Application in yogurt, maintaining pH, acidity, transparency, and functional potential; stability for 11 months	[114]
EPA and DHA	Fish oil	Surfactants: Tween 20 + SDS + lecithin; antioxidant: rosemary extract	High-pressure homogenization	~175 nm	Oxidative stability increased up to 3× with rosemary extract; stability for 11 weeks at 25 °C	[99]
ALA	Flaxseed oil	Sucrose ester (emulsifier) + purified water (aqueous continuous phase)	High-energy homogenization	674–799 nm	Good stability after freeze–thaw cycles; ↑ plasma EPA and DHA levels in rats; sensory acceptability enhanced by the nanostructured formulation	[112]
EPA, DHA, Omega-6, and balanced PUFA mixtures	Mixtures of vegetable oils (olive + palm olein) + krill oil (MKO) or flaxseed oil (MLO)	Whey protein concentrate (WPC) + maltodextrin (MD) + arabic gum (GA), at a ratio of 8:2:1	Pre-homogenization with Ultra-Turrax + Microfluidization or Ultrasound	~198.5 nm (MKO by US); ~201.3 nm (MKO by MF); ~824.9 nm (MLO by US); ~714.2 nm (MLO by MF)	Higher encapsulation efficiency with microfluidization + spray-drying (EE > 85%); improved oxidative stability and more spherical morphology with spray-drying	[111]
ALA, EPA and DHA	Fish/vegetable oil	Surfactant: Laureth-21; Co-surfactant: PEG-40 hydrogenated castor oil (HCO-40)	SNEDDS (self-nanoemulsifying drug delivery system) + pseudo-ternary phase diagram	71–195 nm	High encapsulation (43–87%); greater release and permeation vs. tablet and suspension; ↓ ulceration in rats	[104]
DHA	Refined fish oil	Tween 80, Span 80 (surfactants) + whey protein isolate (WPI)	High-pressure homogenization	120–180 nm	High encapsulation efficiency (~90%); stable under pH, salts, and temperature variations; ↑ bioavailability in rats; ↓ lipid peroxidation	[115]
ALA	Flaxseed oil	Different food-grade emulsifiers (Tween 80 was the most effective)	High-pressure homogenization	70–150 nm	Nanoemulsions maintained stable characteristics under refrigerated storage, showing lower lipid oxidation compared with pure oil	[116]
ALA	Flaxseed oil	Food-grade surfactants (mainly Tween 80 and lecithin)	High-pressure homogenization and ultrasound	50–150 nm	Nanoemulsions exhibited good physicochemical stability, higher oxidative resistance, and potential for application in functional beverages and nutritional supplements	[117]
DHA	DHA in triglyceride form (commercial)	Egg phosphatidylcholine (EPC), cholesterol (CHOL), DSPE-PEG2000-FA (when folate-decorated)	Thin-film solvent evaporation followed by microfluidizer processing	~157.7 nm (PTX/DHA-LNs); ~186.6 nm (PTX/DHA-FA-LNs, folate-decorated)	High encapsulation efficiency (EE > 90%); stable in PBS and serum for 24 h; controlled release without burst effect (100% in 48 h); enhanced folate receptor-mediated internalization and improved antitumor efficacy in mice	[118]

↑ = increasead; ↓ = decreased.

**Table 3:** Encapsulation of Omega Fatty Acids in Liposomes.

Type of Omega	Source of Omega	Liposomal Composition	Production Method	Size/EE%	Additional Remarks	References
EPA and DHA	Fish oil	Liposome + nanoemulsion (SMEDS)	High-shear homogenization and softgel encapsulation	100–300 nm	13.2-fold (EPA) and 4.7-fold (DHA) increase in bioavailability in rats compared with conventional fish oil; low oxidation for 6 months	[137]
EPA and DHA	Cod liver oil and shrimp lipid extract, and carp FPH	Soy lecithin + FO (cod) + shrimp extract + FPH; coating with CS/WPC (mono-, bi-, or composite layer)	Ultrasonication, layer-by-layer coating (CS and WPC), and lyophilization	38.1–100 nm/92.8–97.7%	Better oxidative stability in bilayer nanoliposomes (3 months); controlled release (low in stomach and high in intestine); 1.5 g of powder in 100 g of milk supplied daily PUFA and amino acid requirements with good sensory acceptance (fishy odor and taste masked)	[139]
EPA and DHA	Fish oil	Soy lecithin + curcumin (ethanolic extract) + omega-3 (1:4, 1:8, 1:12, 1:16 extract:lecithin ratio)	Dissolution of extract in ethanol, addition to acetate buffer, homogenization and ultrasonication	100–170 nm/>50%	Controlled release in simulated gastrointestinal medium; ↑ antioxidant activity in formulations with higher curcumin + omega-3 content; ↑ antimicrobial activity	[134]
EPA and DHA	Shrimp oil	Soy phosphatidylcholine (2.5%) + cholesterol + enriched shrimp oil (2%) + glycerol (2% *v*/*v*)	Dissolution in heated ethanol, oil addition, hydration in water + glycerol, ultrasonication, solvent removal, and lyophilization	~170–200 nm/97.6%	↑ oxidative stability during 25 days of storage; good sensory acceptance; free fatty acid permeation in Caco-2 cells reduced from 85% to 75%, demonstrating modulation of absorption	[146]
ALA	Chia oil	Soy phosphatidylcholine + Tween 80 + chia oil + LA/hydroxypropyl-β-cyclodextrin inclusion complex	Thin-film hydration + probe sonication	~52.2 nm/80.2% (LA); 76.4% (chia oil/ALA)	Applied in fortified cow’s milk, providing per serving (240 mL) 236 mg LA and 720 mg ALA; stable for 7 days at 4 °C; remained sensorially acceptable	[135]
EPA and DHA	Pistachio oil	Pistachio oil + lecithin + PEG (PEGylated nanoliposomes)	Oil + lecithin, sonication, and nanoliposomal suspension formation	100–250 nm	↑ in serum EPA and DHA levels in clinical trial patients, with consequent ↓ of inflammatory cytokines and MMP-9, ↑ IL-4, IL-5, and IL-10; no severe adverse events	[147]
EPA and DHA	Fish oil	Salmon lecithin + PUFAs + coating with chitosan/gelatin blend	Lecithin hydration (2%), oil addition (1:10, 1:5, 1:2), sonication, and coating with chitosan/gelatin (0.3:0.1 or 0.2:0.2)	Without coating: ~209–491 nm/62.9–74.5%; coated (SDNLs): ~420–454 nm/81.6%	Coating enhanced thermal and oxidative stability, acting as a physical and antioxidant barriers	[142]
EPA and DHA	Skipjack tuna eye oil	Soy lecithin (1–5%) + EPA/DHA-enriched oil (1–5%); addition of glycerol (2% *v*/*v*) as stabilizer	Hydration in ethanol, evaporation, nanoliposome formation, ultrasonication	Without ultrasonication: 22.8 nm/88%; with ultrasonication: 31–67 nm/98%	Ultrasonication ↑ encapsulation efficiency and the average particle size; fortification in pasteurized milk with 2.5% NL maintained sensory acceptance and ↑ PUFA content in milk; good oxidative stability	[144]
ALA	Chia oil	Phospholipid fraction rich in PI, PA, PE, PG, PC, and lyso-PC, obtained from the polar residue of chia oil	Folch extraction + spontaneous lipid hydration + sonication	~118 nm	Transformation of phospholipid-rich byproducts (extraction residue) into functional nanocarriers	[145]
EPA and DHA	Fish oil	Soy lecithin + cholesterol + brown and green macroalgae extracts	Thin-film lipid hydration + sonication + lyophilization	129–266 nm/99.9%	Nanoliposomes strongly ↓ lipid oxidation; good color stability and controlled release profile (<35%); comparable to or better than synthetic antioxidant (BHT)	[133]
EPA and DHA	Fish oil	Omega-3-rich phosphatidylcholine + cholesterol (PEGylated nanoliposome formation)	Lipid film hydration + sonication	90–120 nm	Resistance to simulated gastric fluid; intestinal absorption confirmed in Caco-2 cells; anti-inflammatory effect in colitis model; safety demonstrated in cells, blood, and mice	[138]
EPA, DHA, and linoleic acid	Fish oil (EPA/DHA) + linoleic acid	Phosphatidylcholine + cholesterol, co-encapsulating PUFAs + curcumin; surface functionalized with chitosan and whey protein	Thin-film hydration + sonication, followed by CH/WPI coating	150–200 nm/85% for curcumin and PUFAs	Chitosan and whey protein coating enhanced oxidative and thermal stability, improved water solubility and oral bioavailability, and enabled co-delivery of PUFAs + antioxidant (curcumin)	[143]

Notes: EE% = Encapsulation Efficiency (%); ↑ = increasead; ↓ = decreased.

**Table 4:** Classes of encapsulating biomaterials and their technological applications.

Types	Subtypes	Examples	Properties	Applications
Biopolymeric	Polysaccharides	Arabic Gum	High solubility, good emulsification	Supplements, drinks
		Pectin	Pectins are natural polysaccharides, formed by galacturonic acid chains, and are widely used as gelling and encapsulating agents in the food and pharmaceutical industries	Gastrointestinal capsules
		Sodium alginate	Forms ionic gels with Ca^2+^	Gastrointestinal capsules
		Modified starch	Improves acid resistance and enzymatic digestion, good oxidative stability and solubility	Gastrointestinal capsules
		Carrageenan	Gel formation, compatible with controlled release	Gastrointestinal capsules
		Maltodextrin	Spray-drying matrix former	Gastrointestinal capsules
	Cellulosic	Ethylcellulose	Coating agent for tablets and capsules, providing controlled drug release	Encapsulating agent in supplement formulations
		Hydroxypropyl- methylcellulose	Cellulose derivative with methyl and hydroxypropyl group substitutions	Modified-release capsules
	Proteins	Gelatin	Forms thermoreversible gels and is a good emulsifier	Softgels and capsules
		Casein	Good interaction with lipids	Electrostatic complexes between casein and anionic polysaccharides
		Soy protein	Good oil retention	Functionality comparable to casein
Lipids		Lecithin	More effective encapsulating agents for the nanoencapsulation of omegas, due to their ability to stabilize oil/water interfaces and form self-organized nanometric structures.	Liquid supplements and softgels
		Mono/di-glycerides	Amphiphilic compounds are auxiliary agents in the encapsulation of omega family fatty acids	They help in the formation of dry microcapsules with proteins, polysaccharides, and modified starches, etc.
Synthetic biodegradable polymers		Poly-lactic-co-glycolic acid	Extended release and biodegradable, resulting in natural products (lactic acid and glycolic acid)	Microparticles and nanoparticles are used to encapsulate medicines and dietary supplements, promoting sustained release
		Polycaprolactone	Synthetic semicrystalline polyester, biodegradable and biocompatible	Targeted capsules and microcapsules
		Polyethylene glycol	It is a hydrophilic, non-ionic polymer of the polyol family	Targeted capsules
Auxiliary functional compounds		Natural antioxidants (e.g., tocopherols, ascorbic acid)	Act by interrupting free radical chain reactions, stabilizing lipids during processing and storage	Reduce the oxidation of omegas during and after encapsulation

## Data Availability

No new data were created or analyzed in this study.

## Abbreviations

The following abbreviations are used in this manuscript:

-COOH	Carboxyl
SFA	Saturated fatty acids
MUFA	Monounsaturated fatty acids
PUFA	Polyunsaturated fatty acids
n-3	Ômega 3
n-6	Ômega 6
n-9	Ômega 9
LC-PUFA	Long-chain polyunsaturated fatty acids
ALA	Alpha-linolenic acid
EPA	Eicosapentanoic acid
DHA	Docosahexaenoic acid
LA	Linoleic acid
ARA	Arachidonic acid
ROS	Reactive oxygen species
TAG	Triacylglycerols
MAGs	Monoacylglycerols
FFAs	Free fatty acids
Ees	Ethyl esters
O/W	Oil-in-water
W/O	Water-in-oil
W/O/W	Multiple emulsions
O/W/O	
PDI	Polydispersity index
ULVs	Unilamellar vesicles
MLVs	Multilamellar vesicles
DLS	Dynamic light scattering
GC-HS	Headspace gas chromatography
FTIR	Fourier-transform infrared
UV-Vis	Ultraviolet and visible light
SEM	Scanning electron microscopy
TEM	Transmission electron microscopy
SD	Spray drying
SFD	Spray freeze-drying
FD	Freeze-drying
MFD	Microwave freeze-drying
SLNs	Solid nanolipids
NLCs	Nanostructured lipid carriers
kDa	Kilodalton
IL-6	Interleukin-6
TNF-α	Tumor necrosis factor alpha
COX-2	Cyclooxygenase-2
oxLDL	Oxidized low-density lipoprotein
PPARs	Peroxisome proliferator-activated receptors
FDA	Food and Drug Administration
EMA	European Medicines Agency
ICH	International Council for Harmonization
HPH	High-pressure homogenization
MKO	Krill oil
MLO	Flaxseed oil
WPC	Whey protein concentrate
MD	Maltodextrin
GA	Arabic gum
HCO-40	Hydrogenated castor oil
SNEDDS	Self-nanoemulsifying drug delivery system
WPI	Whey protein isolate
EPC	Egg phosphatidylcholine
CHOL	Cholesterol
PBS	Phosphate-buffered saline
SMEDS	Self-micro-emulsifying delivery system
IL-4	Interleukin-4
IL-5	Interleukin-5
IL-10	Interleukin-10
PASI	Psoriasis Area and Severity Index
HBL	Hydrophilic–Lipophilic Balance
SDS	Sodium dodecyl sulfate
US	Ultrasound
MF	Microfluidization
PTX	Paclitaxel
LN	Lipid nanoemulsion
FA	Folic acid
FO	Fish oil
FPH	Fish protein hydrolysate
CH	Chitosan
MMP-9	Matrix Metalloproteinase-9
NL	Nanoliposomes
PI	Phosphatidylinositol
PA	Phosphatidic acid
PE	Phosphatidylethanolamine
PG	Phosphatidylglycerol
PC	Phosphatidylcholine
Lyso-PC	Lysophosphatidyl-choline
